# Kaposi's sarcoma herpesvirus (KSHV) microRNA K12-1 functions as an oncogene by activating NF-κB/IL-6/STAT3 signaling

**DOI:** 10.18632/oncotarget.9221

**Published:** 2016-05-07

**Authors:** Mingqing Chen, Fan Sun, Lei Han, Zhaoxia Qu

**Affiliations:** ^1^ University of Pittsburgh Cancer Institute, University of Pittsburgh School of Medicine, Pittsburgh, PA, USA; ^2^ Department of Microbiology and Molecular Genetics, University of Pittsburgh School of Medicine, Pittsburgh, PA, USA; ^3^ Hubei Key Laboratory of Genetic Regulation and Integrative Biology, School of Life Sciences, Central China Normal University, Wuhan, Hubei, China

**Keywords:** KSHV/HHV8, miR-K12-1, NF-κB, IL-6, STAT3

## Abstract

The human oncogenic virus Kaposi's sarcoma herpesvirus (KSHV) is the most common cause of malignancies among AIDS patients. KSHV possesses over hundred genes, including 25 microRNAs (miRNAs). The roles of these miRNAs and many other viral genes in KSHV biology and pathogenesis remain largely unknown. Accordingly, the molecular mechanisms by which KSHV induces tumorigenesis are still poorly defined. Here, we identify KSHV miRNA K12-1 (miR-K12-1) as a novel viral oncogene by activating two important transcription factors, nuclear factor-κb (NF-κB) and signal transducer and activator of transcription 3 (STAT3). Interestingly, miR-K12-1 activates STAT3 indirectly through inducing NF-κB activation and NF-κB-dependent expression of the cytokine interleukin-6 (IL-6) by repressing the expression of the NF-κB inhibitor IκBα. Accordingly, expression of ectopic IκBα or knockdown of NF-κB RelA, IL-6 or STAT3 prevents expression of cell growth genes and suppresses the oncogenicities of both miR-K12-1 and KSHV. These data identify miR-K12-1/NF-κB/IL-6/STAT3 as a novel oncogenic signaling underlying KSHV tumorigenesis. These data also provide the first evidence showing that IL-6/STAT3 signaling acts as an essential mediator of NF-κB oncogenic actions. These findings significantly improve our understanding of KSHV pathogenesis and oncogenic interaction between NF-κB and STAT3.

## INTRODUCTION

Kaposi's sarcoma herpesvirus (KSHV), also known as human herpesvirus 8 (HHV8), is the etiological agent of three malignancies mainly associated with acquired immunodeficiency syndrome (AIDS) and immunosuppression: Kaposi's sarcoma (KS), primary effusion lymphoma (PEL) and multicentric Castleman's disease (MCD) [1–3]. Although the molecular biology of this oncogenic virus has been extensively studied during the past two decades, the mechanisms by which KSHV induces tumorigenesis still remain obscure [4]. Those characterized to date suggest that while its lytic infection contributes to tumor promotion in a paracrine manner, the latent infection of KSHV is the direct driving force in tumor formation and maintenance with the expression of a limited set of viral genes capable of hijacking cellular signaling pathways important for host cell proliferation and survival [5].

During latent infection, KSHV also expresses 12 pre-microRNAs (pre-miRNAs) that are processed to yield 25 mature miRNAs [6]. Mature miRNAs are approximately 22 nucleotides long, single-stranded non-coding RNAs that bind to mRNAs, resulting in their degradation and/or translational repression [7, 8]. Bioinformatic searches using the seed sequences of miRNAs reveal a large numbers of potential viral miRNA targets. However, so far only a few of them have been validated to be true targets of viral miRNAs [4]. Accordingly, the roles of those viral miRNAs in KSHV biology and pathogenesis remain largely unknown.

One KSHV miRNA, miRNA K12-1 (miR-K12-1), was reported to directly target and repress the expression of the cellular protein IκBα, the primary inhibitor of the transcription factor nuclear factor-κb (NF-κB) [9, 10]. NF-κB plays a causative role in the formation and therapeutic resistance of several tumor types, including those induced by KSHV [10–12]. Whereas its transient or low activation may contribute to KSHV lytic replication, constitutive hyper-activation of NF-κB plays a crucial role in the latency of KSHV as well as in the formation and maintenance of KSHV-associated tumors. Inhibition of NF-κB activation by chemical inhibitors or short hairpin RNAs (shRNAs) against the NF-κB activating kinase IKK induces reactivation of KSHV from latency, prevents human endothelial cell transformation by KSHV, and reverses malignant phenotype and tumorigenicity of KSHV-associated PEL cells both *in vitro* and *in vivo* [13–17]. Similarly, NF-κB specific inhibition by over-expressing a functional defective mutant of RelA (also known as p65), the prototypical member of the NF-κB family, or specific RelA shRNAs results in reactivation of latent KSHV and growth inhibition in PEL cells [5, 17].

The transcription factor signal transducer and activator of transcription 3 (STAT3) is another important cellular protein involved in the tumorigenesis of KSHV [5]. Like the inhibition of NF-κB, STAT3 inhibition by its dominant-negative mutant, pharmacological inhibitor or shRNAs results in death of KSHV-associated PEL cells [5, 18, 19]. However, it remains unknown whether and how these two key pro-tumorigenic factors crosstalk in KSHV tumorigenesis.

In this study, we demonstrate that miR-K12-1 functions as an oncogene by hijacking IκBα/NF-κB signaling to induce interleukin-6 (IL-6)-dependent activation of STAT3. We also demonstrate that the novel miR-K12-1/IκBα/NF-κB/IL-6/STAT3 oncogenic signaling pathway is an essential mechanism underlying KSHV tumorigenesis.

## RESULTS

### KSHV miR-K12-1 activates both NF-κB and STAT3 and functions as an oncogene

**Figure 1:**
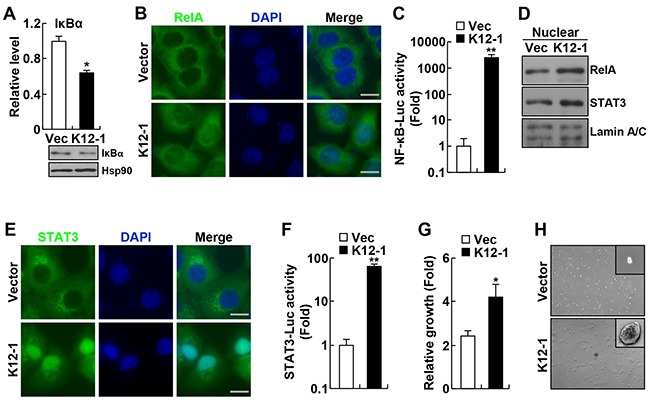
KSHV miR-K12-1 induces NF-κB and STAT3 activation and cell transformation **A.** miR-K12-1 represses RNA and protein expression of IκBα. Real-time RT-PCR and immunoblotting analyses were performed to examine the IκBα RNA and protein levels, respectively, in Hela cells transfected with miR-K12-1 or an empty vector. **B.** miR-K12-1 induces RelA nuclear translocation. Immunofluorescence assay was performed to examine the nuclear translocation of RelA protein in Hela cells transfected with miR-K12-1 or an empty vector. Scale bar: 10 μm. **C.** miR-K12-1 induces transcriptional activation of NF-κB. Luciferase assay was performed to measure NF-κB transcriptional activity in Hela cells transfected with miR-K12-1 or an empty vector, together with NF-κB luciferase reporter. **D.** miR-K12-1 induces RelA and STAT3 nuclear expression. Nuclear fraction immunoblotting was performed to examine the nuclear expression of RelA and STAT3 proteins in Hela cells transfected with miR-K12-1 or an empty vector. **E.** miR-K12-1 induces STAT3 nuclear translocation. Immunofluorescence assay was performed to examine the nuclear translocation of STAT3 protein in Hela cells transfected with miR-K12-1 or an empty vector. Scale bar: 10 μm. **F.** miR-K12-1 induces transcriptional activation of STAT3. Luciferase assay was performed to measure STAT3 transcriptional activity in Hela cells transfected with miR-K12-1 or an empty vector, together with STAT3 luciferase reporter. **G.** miR-K12-1 promotes cell growth. Cell growth rates were determined for Rat-1 cells stably expressing miR-K12-1 or an empty vector. **H.** miR-K12-1 induces cell transformation. Soft agar colony formation assay was performed for Rat-1 cells stably expressing miR-K12-1 or an empty vector.

To investigate the significance of miR-K12-1 in KSHV oncogenicity, we first examined whether K12-1 represses the expression of IκBα in our system. In line with previous studies [9], we found that expression of miR-K12-1 repressed the expression of IκBα at both RNA and protein levels (Figure 1A). Following these studies, we examined whether miR-K12-1 induces nuclear translocation of RelA and NF-κB transcriptional activation. The role of IκBα is to sequester RelA and other NF-κB members in the cytoplasm, thereby preventing NF-κB transcriptional activation [10]. As expected, miR-K12-1 induced strong nuclear translocation of RelA and transcriptional activation of NF-κB (Figure 1B–1D).

Interestingly, we found that miR-K12-1 is also a strong activator of STAT3, because miR-K12-1 induced strong nuclear translocation and transcriptional activation of STAT3 (Figure 1D–1F). Like NF-κB, STAT3 is an abundant latent cytoplasmic transcription factor that can translocate into the nucleus to induce gene transcription after being activated, although they are regulated by entirely different signaling mechanisms [20]. Also like NF-κB, STAT3 is often constitutively activated in many human cancers, including those induced by KSHV [5, 21].

Given the tumor-promoting role of both NF-κB and STAT3 activations, we hypothesized that miR-K12-1 is a viral oncogene that was not identified previously. In support of this hypothesis, we found that expression of miR-K12-1 significantly increased cell growth even in normal culture condition (Figure 1G). To directly test the hypothesis, we examined whether miR-K12-1 induces colony formation of the Rat-1 fibroblast cells in soft agar, a model system that has been widely used to test the oncogenic potential of candidate genes, particularly viral genes [22–25]. As expected, Rat-1 cells stably expressing an empty control vector did not form colonies in soft agar (Figure 1H, and Supplementary Figure S1). However, expression of miR-K12-1 alone was sufficient to induce Rat-1 cells to form colonies in soft agar. These data suggest that miR-K12-1 indeed has neoplastic transforming ability and functions as an oncogene. These data also suggest that the oncogenic potential of miR-K12-1 is associated with its ability in activating NF-κB and STAT3.

### KSHV miR-K12-1 activates STAT3 indirectly through targeting the IκBα/NF-κB/IL-6 signaling pathway

To investigate the molecular mechanisms by which miR-K12-1 induces STAT3 activation, we analyzed whether miR-K12-1 targets the negative regulators of STAT3, such as suppressor of cytokine signaling 3 (SOCS3), protein inhibitor of activated STAT3 (PIAS3) or protein tyrosine phosphatases (PTPs). However, our bioinformatics analysis using miRNA target prediction software suggested that miR-K12-1 did not target those STAT3 negative regulators or any other known inhibitors of STAT3 signaling (data not shown), suggesting that miR-K12-1 activates STAT3 indirectly through activating STAT3 upstream activator(s) by repressing their inhibitor(s).

**Figure 2:**
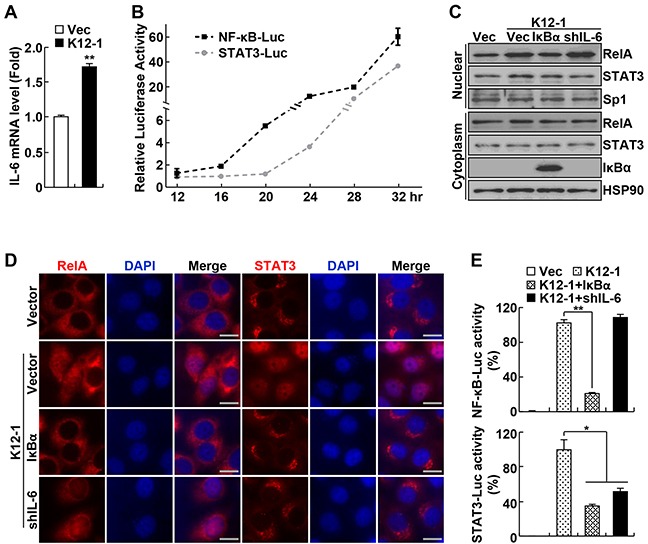
KSHV miR-K12-1 activates STAT3 via activating IκBα/NF-κB/IL-6 signaling **A.** miR-K12-1 induces IL-6 expression. Real-time RT-PCR analysis was performed to examine the RNA levels of IL-6 in Hela cells transfected with miR-K12-1 or an empty vector. **B.** miR-K12-1 induces a delayed STAT3 activation compared to NF-κB activation. The dynamics of NF-κB and STAT3 transcriptional activation induced by miR-K12-1 were determined using luciferase assay for Hela cells co-transfected with miR-K12-1 and NF-κB or STAT3 luciferase reporter, respectively. **C.** IκBα expression or IL-6 knockdown inhibits STAT3 nuclear expression induced by miR-K12-1. Subcellular fraction immunoblotting was performed to examine the nuclear expression of RelA and STAT3 proteins in Hela cells transfected with the indicated constructs. **D.** IκBα expression or IL-6 knockdown prevents STAT3 nuclear translocation induced by miR-K12-1. Immunofluorescence assay was performed to examine the nuclear translocation of RelA and STAT3 proteins in Hela cells transfected with the indicated constructs. Scale bar: 10 μm. **E.** IκBα expression or IL-6 knockdown represses STAT3 transcriptional activation induced by miR-K12-1. Luciferase assay was performed to measure NF-κB and STAT3 transcriptional activity in Hela cells transfected with the indicated constructs together with NF-κB or STAT3 luciferase reporter, respectively.

In this regard, we examined whether miR-K12-1 induces expression of the cytokine IL-6, a transcriptional target of NF-κB that functions as a potent STAT3 activator [26]. Indeed, miR-K12-1 induced IL-6 expression (Figure 2A). We then compared the activation dynamics of NF-κB and STAT3 induced by miR-K12-1, and found a four-hour delay in STAT3 activation in comparison to NF-κB activation (Figure 2B). These data suggested that the miR-K12-1 oncogene induces STAT3 activation through activating NF-κB and NF-κB-dependent IL-6 induction.

To validate this, we examined the effects of IκBα expression and IL-6 knockdown on miR-K12-1 activation of NF-κB and STAT3. Expression of ectopic IκBα blocked miR-K12-1-induced nuclear translocation and transcriptional activation of both NF-κB RelA and STAT3 (Figure 2C–2E). On the other hand, shRNA knockdown of IL-6 only prevented STAT3 activation but had no effect on NF-κB activation by miR-K12-1. It should be pointed out that IκBα expression or IL-6 knockdown had no effect on the expression of transfected miR-K12-1 (Supplementary Figure S2). Consistent with its role in suppressing STAT3 activation by IL-6, SOCS3 over-expression inhibited STAT3 but not NF-κB activation induced by miR-K12-1 (Supplementary Figure S3). These data together indicate that the viral oncogene miR-K12-1 activates STAT3 through targeting IκBα and subsequent NF-κB activation and IL-6 induction.

### The oncogenicity of miR-K12-1 requires IκBα repression and NF-κB/IL-6/STAT3 activation

**Figure 3:**
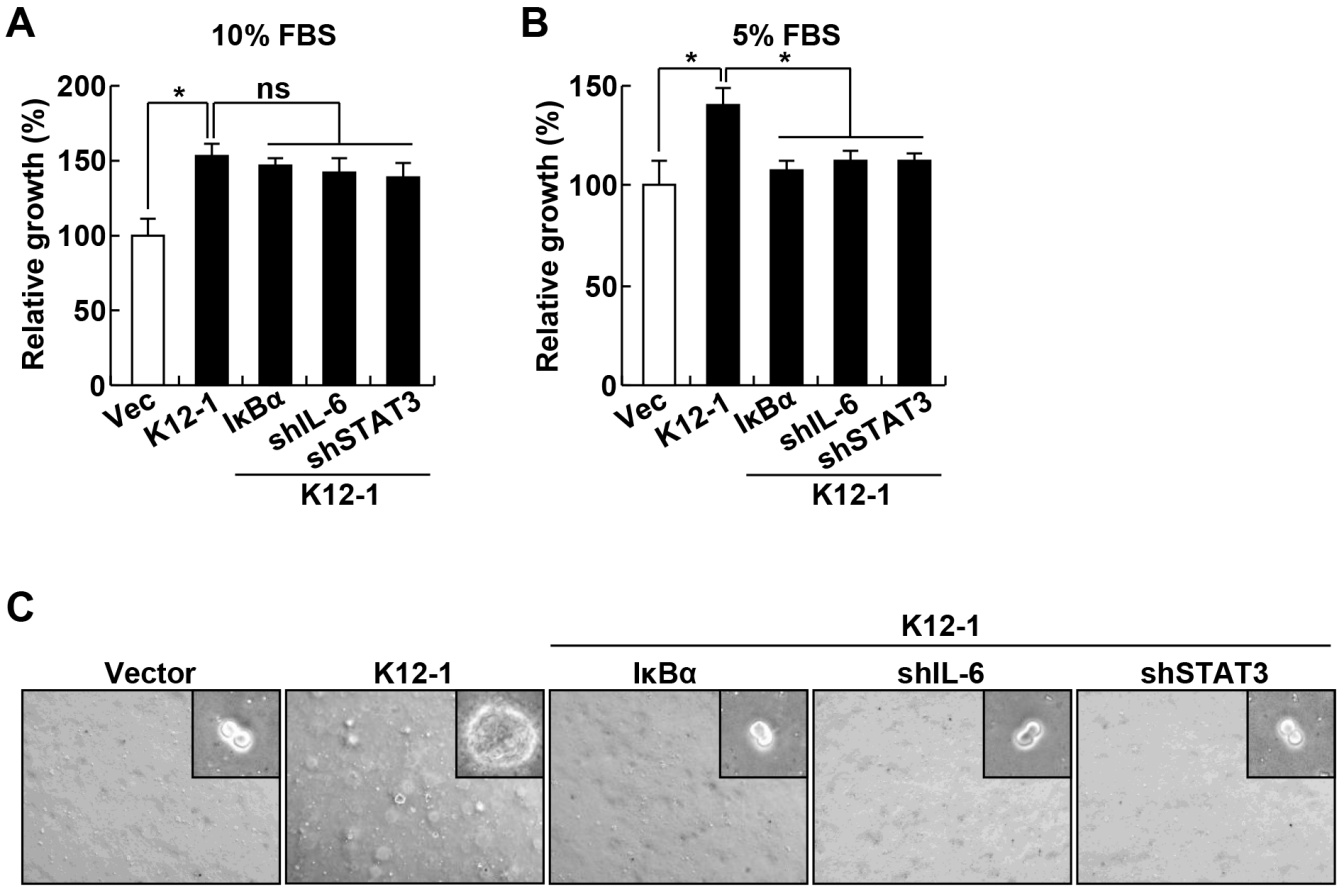
The oncogenicity of KSHV miR-K12-1 depends on the activation of IκBα/NF-κB/IL-6/STAT3 signaling **A.** IκBα expression, IL-6 knockdown, or STAT3 knockdown has no obvious effect on miR-K12-1-expressing cells cultured in medium containing 10% FBS. Cell growth rates were determined for Rat-1 cells stably expressing an empty vector, miR-K12-1, or miR-K12-1 together with IκBα, IL-6 shRNA or STAT3 shRNA cultured in medium containing 10% FBS. **B.** IκBα expression, IL-6 knockdown, or STAT3 knockdown suppresses miR-K12-1-driven cell growth when the supplemented FBS is decreased to 5%. Cell growth rates were determined for Rat-1 cells stably expressing an empty vector, miR-K12-1, or miR-K12-1 together with IκBα, IL-6 shRNA or STAT3 shRNA cultured in medium containing 5% FBS. **C.** IκBα expression, IL-6 knockdown, or STAT3 knockdown suppresses miR-K12-1-driven cell transformation. Soft agar colony formation assay was performed for Rat-1 cells stably expressing an empty vector, miR-K12-1, or miR-K12-1 together with IκBα, IL-6 shRNA or STAT3 shRNA.

To examine whether miR-K12-1 exerts its oncogenicity through the IκBα/NF-κB/IL-6/STAT3 signaling pathway, we generated miR-K12-1 stable Rat-1 cell lines that also express ectopic IκBα or shRNAs specifically against IL-6 or STAT3. Of note, expression of ectopic IκBα or knockdown of IL-6 or STAT3 did not affect the expression of the viral miRNA, because its expression levels in these stable cell lines were comparable (Supplementary Figure S4). Expression of IκBα or knockdown of IL-6 or STAT3 had no obvious effect on miR-K12-1-driven cell growth when 10% fetal bovine serum (FBS) was provided in cell culture medium (Figure 3A). However, the cell growth driven by miR-K12-1 was almost completely inhibited by IκBα expression when the supplemented FBS was decreased to 5% (Figure 3B). Similar inhibitions were also seen by IL-6 or STAT3 knockdown. More importantly, the neoplastic transforming ability of miR-K12-1 was also completely inhibited by IκBα expression, IL-6 knockdown or STAT3 knockdown (Figure 3C, and Supplementary Figure S5). These data suggest that miR-K12-1 functions as an oncogene via targeting the IκBα/NF-κB/IL-6/STAT3 signaling pathway.

### Activation of miR-K12-1/NF-κB/IL-6/STAT3 signaling is required for KSHV tumorigenesis

**Figure 4:**
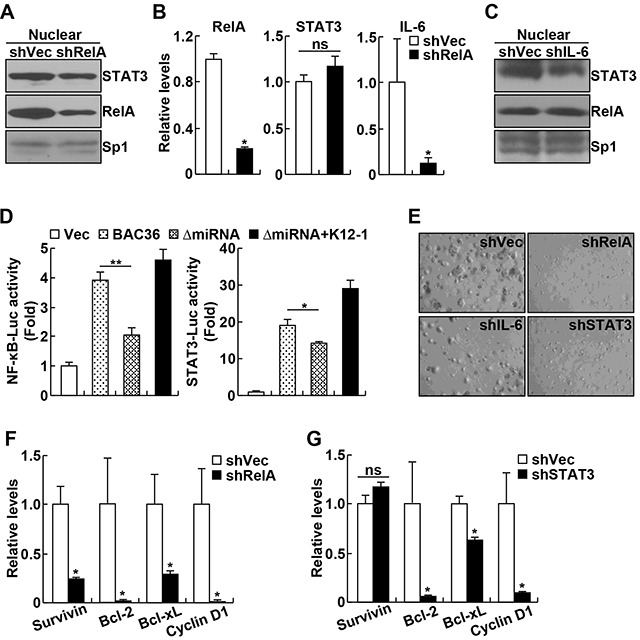
KSHV oncogenicity involves activation of miR-K12-1/NF-κB/IL-6/STAT3 signaling **A.** RelA knockdown decreases STAT3 nuclear expression in BCBL-1 cells. Nuclear fraction immunoblotting assay was performed to examine the nuclear expression of RelA and STAT3 proteins in BCBL-1 cells stably expressing RelA shRNA or an empty vector. **B.** RelA knockdown suppresses IL-6 but not STAT3 expression in BCBL-1 cells. Real time PCR analysis was performed to detect RelA, IL-6 and STAT3 RNA expression levels in BCBL-1 cells stably expressing RelA shRNA or an empty vector. **C.** IL-6 knockdown blocks STAT3 nuclear expression in BCBL-1 cells. Nuclear fraction immunoblotting assay was performed to examine the nuclear expression of RelA and STAT3 proteins in BCBL-1 cells stably expressing IL-6 shRNA or an empty vector. **D.** KSHV requires miR-K12-1 to induce NF-κB and STAT3 activation. Luciferase reporter activity assay was performed to examine the activation of NF-κB and STAT3 in 293FT cells with an empty vector, KSHVBac36, KSHVBac36 ΔmiRNA, or KSHVBac36 ΔmiRNA plus miR-K12-1. **E.** Knockdown of RelA, IL-6 or STAT3 suppresses the oncogenicty of BCBL-1 cells. Soft agar colony formation assay was performed for BCBL-1 cells stably expressing RelA shRNA, IL-6 shRNA, STAT3 shRNA, or an empty vector. **F.** RelA knockdown suppresses expression of cell survival and proliferation genes in BCBL-1 cells. Real time PCR analysis was performed to detect RNA expression levels of the indicated genes in BCBL-1 cells stably expressing RelA shRNA or an empty vector. **G.** STAT3 knockdown suppresses expression of cell survival and proliferation genes in BCBL-1 cells. Real time PCR analysis was performed to detect RNA expression levels of the indicated genes in BCBL-1 cells stably expressing STAT3 shRNA or an empty vector.

We next would like to investigate the pathophysiological relevance of the miR-K12-1/NF-κB/IL-6/STAT3 signaling pathway in KSHV tumorigenesis. We generated KSHV^+^ BCBL-1 PEL cell lines stably expressing shRNAs specifically against RelA, IL-6 or STAT3. Consistent with our recent studies showing high activations of NF-κB and STAT3 in various PEL cell lines [5], our nuclear fraction-immunoblotting (IB) and immunofluorescence assays indicated a strong nuclear expression of RelA and STAT3 in BCBL-1 cells stably expressing scrambled control shRNAs (Figure 4A, and Supplementary Figure S6). Knockdown of RelA significantly inhibited the nuclear expression of STAT3 in BCBL-1 cells. Of note, RelA knockdown had no effect on the STAT3 expression (Figure 4B). As expected, the expression of IL-6 in BCBL-1 cells was largely repressed by RelA knockdown. Interestingly, IL-6 knockdown, like the RelA knockdown, also inhibited STAT3 nuclear expression hence STAT3 activation in BCBL-1 cells (Figure 4C). Consistent with the fact that IL-6 only functions as an effector but not an activator of NF-κB, IL-6 knockdown did not affect RelA nuclear expression in BCBL-1 cells.

To repress miR-K12-1 expression in BCBL-1 cells, we initially used lock-nucleic acid (LNA)-based miR-K12-1 suppressor [9]. However, miR-K12-1 could not be repressed in BCBL-1 cells or other PEL cell lines to a detectable level, because of extremely low transfection of these cells. Thus, we changed the strategy and compared NF-κB and STAT3 activations induced by KSHV Bacterial Artificial Chromosome Clone (KSHVBac36) and its mutant (ΔmiRNA) in which miR-K12-1 and other 13 miRNAs are deleted [9]. As expected, expression of wildtype KSHVBac36 induced activation of both NF-κB and STAT3 (Figure 4D). However, the ΔmiRNA mutant lost the ability to activate either NF-κB or STAT3. More interestingly, re-expression of miR-K12-1 could efficiently rescue the ability of the ΔmiRNA mutant in activating NF-κB and STAT3. These data indicate that miR-K12-1/NF-κB/IL-6/STAT3 signaling is activated under KSHV pathogenic condition.

To further validate and also expand these studies, we also investigated the significance of the NF-κB/IL-6/STAT3 signaling pathway in KSHV oncogenesis. To this end, we examined the effect of RelA, IL-6 or STAT3 knockdown on the colony formation of BCBL-1 cells. In line with our recent studies [5], BCBL-1 cells expressing scrambled control shRNAs showed high oncogenicity, as evidenced by their high colony formation ability in soft agar (Figure 4E, and Supplementary Figure S7). However, the colony formation ability of BCBL-1 cells was significantly suppressed by RelA, IL-6 or STAT3 knockdown. In fact, knockdown of RelA, IL-6 or STAT3 was able to block the outgrowth of BCBL-1 cells in normal culture (Supplementary Figure S8). Highly consistent with these data, knockdown of RelA or STAT3 led to significant decreases in the expression of cell survival and proliferation genes, including Bcl-2, Bcl-xL, Survivin and/or Cyclin D1 (Figure 4F–4G). It should be pointed out that although the expressions of Bcl-2, Bcl-xL and Cyclin D1 in BCBL-1 cells were repressed by both RelA and STAT3 knockdowns, which have similar knockdown efficiency (Figure 4B and data not shown), the repression caused by RelA knockdown was stronger. Furthermore, Survivin, like IL-6, was only repressed by RelA but not STAT3 knockdown. These data suggest that activation of NF-κB/IL-6/STAT3 signaling is an important mechanism underlying KSHV oncogenesis. These data also suggest that in addition to activating STAT3 for gene induction, NF-κB also directly induces gene expression contributing to KSHV tumorigenesis.

## DISCUSSION

KSHV encodes over 115 genes including 25 miRNAs [6]. However, the roles of most of these genes, particularly the viral miRNAs, in KSHV biology and pathogenesis remain unclear. The data presented here demonstrate miR-K12-1 as the first viral miRNA that possesses the oncogenic ability, and identify miR-K12-1/IκBα/NF-κB/IL-6/STAT3 as a novel oncogenic signaling pathway important for KSHV tumorigenesis (Supplementary Figure S9). These data also provide the first evidence showing that IL-6/STAT3 signaling acts as an essential downstream mediator of NF-κB oncogenic actions. These findings significantly improve our understanding of KSHV pathogenesis and oncogenic interaction between NF-κB and STAT3. These findings also set up a solid basis for us to target the miR-K12-1/IκBα/NF-κB/IL-6/STAT3 oncogenic signaling pathway for the prevention and treatment of KSHV-mediated cancers, for which no effective therapies exist currently.

Since their original cloning and identification in 2005, a role of KSHV miRNAs in viral pathogenesis has been expected. The miRNAs form a cluster that is located within the genomic region associated with KSHV latency and share the common promoter with several well-known latent genes, including the viral oncoprotein vFLIP [27–31]. Indeed, a virus mutant in which the viral miRNA cluster is deleted not only loses the transforming ability, but actually induces cell cycle arrest and apoptosis [32]. On the other hand, expression of the miRNA cluster decreases mitochondria biogenesis and induces aerobic glycolysis, a metabolic hallmark of cancer cells [33]. Interestingly, the metabolic shift is important for viral latency maintenance and provides a growth advantage. Moreover, several viral miRNAs have been shown to stabilize viral latency or to promote cell growth, migration and/or invasion [34–45]. However, whether any of viral miRNAs function as an oncogene has yet to be validated. Moreover, downstream functional targets of viral miRNAs remain largely unknown. Although recent comprehensive target analyses reveal nearly 1000 potential viral miRNA target genes, so far only a few of them have been validated to be true targets of viral miRNAs [4, 46–48]. Our studies not only validate IκBα as a bona fide target gene of miR-K12-1, but also demonstrate that miR-12-1 functions as an oncogene by targeting IκBα. In addition to IκBα, p21 and caspase-3 have also been suggested to be target genes of miR-K12-1 [49, 50]. Although it is interesting to test whether this function of miR-K12-1 is required for its oncogenic ability, our studies suggest that targeting these two and any other potential genes by miR-K12-1 is not sufficient to induce cell transformation.

Activation of NF-κB/IL-6/STAT3 signaling by miR-K12-1 is a critical mechanism underlying KSHV tumorigenesis, however, KSHV also uses other mechanisms to facilitate the activation of this pro-oncogenic signaling pathway. For example, the viral oncoproein vFLIP encoded by KSHV induces proteasomal degradation of IκBα, complementing miR-K12-1 in NF-κB activation [51]. It will be of interest to examine whether vFLIP is able to activate STAT3, and if so, whether through NF-κB and NF-κB-induced IL-6. In addition to inducing IκBα protein degradation to activate NF-κB, which is called ‘canonical NF-κB activation’, vFLIP also induces activation of the non-canonical NF-κB by inducing processing of p100, atypical NF-κB inhibitor that also serves as the precursor of the mature form of an important NF-κB member NF-κB2 p52 [52, 53]. Both NF-κB pathways have been linked to various cellular and viral tumorigenesis and often crosstalk with each other and other signaling pathways important to cancer pathogenesis, such as autophagy [25, 54–57]. It thus will be interesting to examine whether and how these important signaling pathways interact with each other in the tumorigenesis mediated by miRNA-K12-1, vFLIP or KSHV.

## MATERIALS AND METHODS

### Plasmids and reagents

KSHVBac36, KSHVBac36 ΔmiRNA, and KSHV miR-K12-1 expressing vector were kind gifts from Drs. Paul Lieberman and Rolf Renne. Vectors expressing shRNA against IL-6, RelA, or STAT3 (target sites are listed in Supplementary Table S1), IκBα, SOCS3, NF-κB–driven firefly luciferase reporter, and TK-driven Renilla luciferase reporter have been described before [5, 58]. The Sp1, Lamin A/C, and Hsp90 antibodies as well as the secondary antibodies were from Santa Cruz Biotechnology (Dallas, TX). The RelA and STAT3 antibodies were from Cell Signaling Technology (Danvers, MA). The anti-Myc antibody was generated from the 9E10 hybridoma as described [59].

### Cell culture

Human PEL cell line BCBL-1 cells were maintained in suspension in RPMI 1640 medium supplemented with 10% FBS. Human cell lines Hela and 293FT and rat fiborblast Rat-1 cells were maintained in DMEM medium supplemented with 10% FBS.

### Quantitative polymerase chain reaction (qPCR) analysis

Cells were subjected to RNA extraction, RNA reverse transcription and real-time PCR as described [60]. The expression levels of various genes were normalized to that of GAPDH. Primer pairs used for qPCR are listed in Supplementary Table S2.

### Generation of stable transfectants

Rat-1 cells expressing an empty vector, miR-K12-1, or miR-K12-1 together with IκBα, IL-6 shRNA or STAT3 shRNA, and BCBL-1 cells expressing an empty vector, RelA shRNA, IL-6 shRNA, or STAT3 shRNA were generated as described before [5, 61].

### Soft agar assays

Rat-1 (2.5 × 10^5^) or BCBL-1 (5 × 10^4^) cells suspended in culture medium containing 0.6% SeaPlaque low melting agarose were plated on the top of 1% agarose in culture medium as described before [5, 22–25]. Colonies in soft agar were counted 12 days after plating. Each determination was repeated in at least 3 independent experiments.

### Immunofluorescence microscopy

Cells were fixed, permeabilized, and subsequently incubated with the indicated primary antibodies, followed by fluorescent dye-conjugated secondary antibodies [5, 62]. The stained proteins and their subcellular localizations were detected using a fluorescence microscope. The cells were also counterstained with DAPI for nuclear staining.

### Immunoblotting (IB) analysis

Cytosolic and nuclear extracts or whole cell lysates were prepared as described previously [63, 64]. The whole-cell, cytosolic or nuclear extracts were then subjected to SDS-PAGE and immunoblotting using the indicated antibodies. The purity of the obtained cell nuclear fractions was confirmed by the detection of Sp1 or Lamin A/C (nuclear fraction) but no Hsp90 (cytoplasm) in IB assays.

### Luciferase gene reporter assays

The indicated cell lines were transfected with NF-κB–driven firefly luciferase reporter and TK-driven Renilla luciferase reporter. At 24 h posttransfection, dual luciferase activities were measured as we described previously [58].

### Statistical analysis

Data were reported as mean ± standard deviation (SD). As described previously [65], Student's t test (two tailed) was used to assess significance of differences between two groups, and p values < 0.05 and 0.01 were considered statistically significant and highly statistically significant, and indicated by * and **, respectively.

## SUPPLEMENTARY FIGURES AND TABLES


