# Supplementary material for: Kaposi's sarcoma herpesvirus (KSHV) microRNA K12-1 functions as an oncogene by activating NF-κB/IL-6/STAT3 signaling
Source: Oncotarget. 2016 May 7;7(22):33363–73. doi: 10.18632/oncotarget.9221 (PMC5078101; doi:10.18632/oncotarget.9221)
Supplement: Supplementary file 1 [file oncotarget-07-33363-s001.pdf]

SUPPLEMENTARY FIGURES AND TABLES

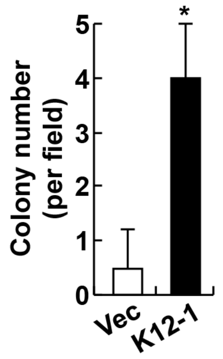

Supplementary Figure S1: Colony numbers of the soft agar colony formation assays in Figure 1H. Cell clusters of three or more cells are counted. Of note, in contrast to miR-K12-1 expressing Rat-1 cells which formed big colonies (Figure 1H), vector-expressing Rat-1 cells only formed small cell clusters of no more than 4 cells.

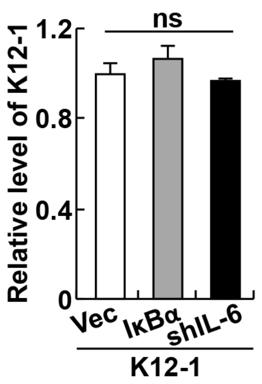

Supplementary Figure S2: IκBα expression or IL-6 knockdown had no effect on the expression of transfected miR-K12-1 in Hela cells. Real-time RT-PCR analysis was performed to examine the expression level of miR-K12-1 in Hela cells transfected with miR-K12-1 together with an empty vector, IκBα, or IL-6 shRNA.

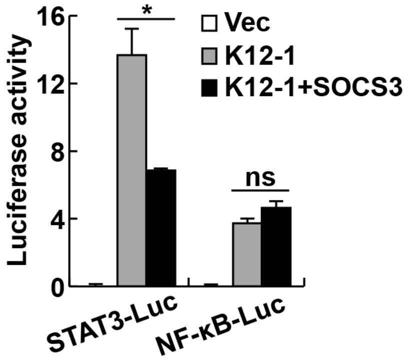

Supplementary Figure S3: SOCS3 inhibits STAT3 but not NF-κB activation induced by miR-K12-1. Luciferase assay was performed to measure STAT3 or NF-κB transcriptional activity in Hela cells transfected with an empty vector, miR-K12-1, or miR-K12-1 plus SOCS3, together with STAT3 or NF-κB luciferase reporter, respectively.

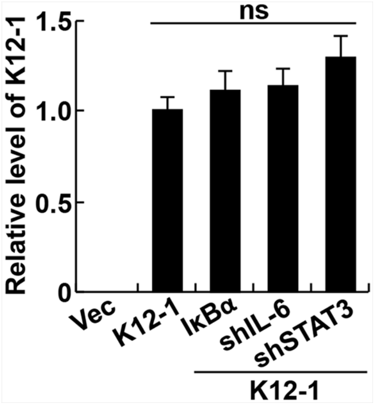

Supplementary Figure S4: Expression of IκBα, or knockdown of IL-6 or STAT3, had no effect on the expression of miR-K12-1 in Rat-1 cells. Real-time RT-PCR analysis was performed to examine the expression level of miR-K12-1 in Rat-1 cells stably expressing an empty vector, miR-K12-1, or miR-K12-1 together with IκBα, IL-6 shRNA, or STAT3 shRNA.

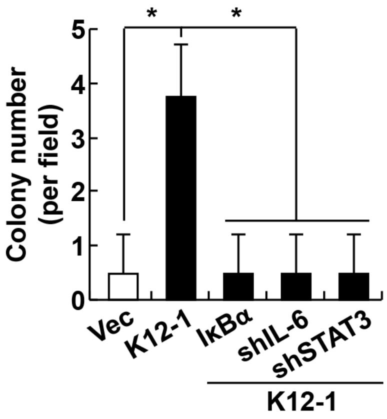

Supplementary Figure S5: Colony numbers of the soft agar colony formation assays in Figure 3C. Cell clusters of three or more cells are counted. Of note, in contrast to Rat-1 cells stably expressing miR-K12-1, which formed big colonies (Figure 3C), Rat-1 cells stably expressing an empty vector, or miR-K12-1 together with IkBa, IL-6 shRNA, or STAT3 shRNA only formed small cell clusters of no more than 4 cells.

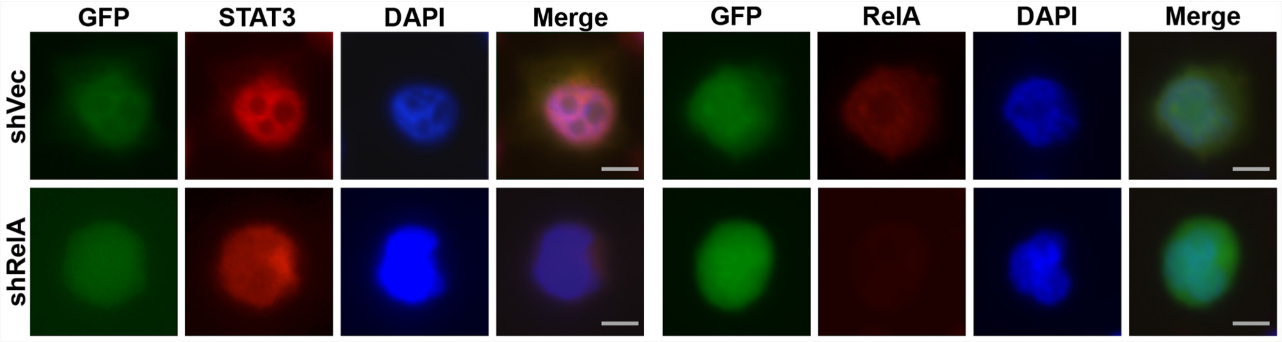

Supplementary Figure S6: Knockdown of RelA inhibits nuclear expression of STAT3 in BCBL-1 cells. Immunofluorescence assay was performed to examine the expression of STAT3 and RelA in BCBL-1 cells stably expressing RelA shRNA or scrambled control shRNA, together with GFP. Scale bar: 5 µm.

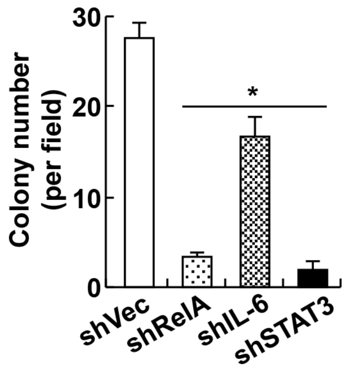

Supplementary Figure S7: Colony numbers of the soft agar colony formation assays in Figure 4E.

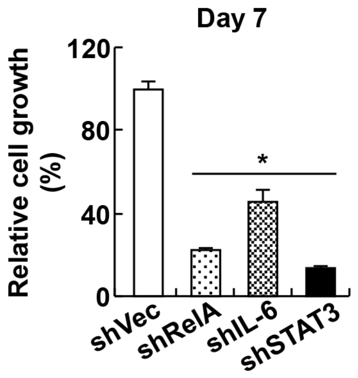

Supplementary Figure S8: Knockdown of RelA, Il-6 or STAT3 blocks BCBL-1 cell growth in cell culture.

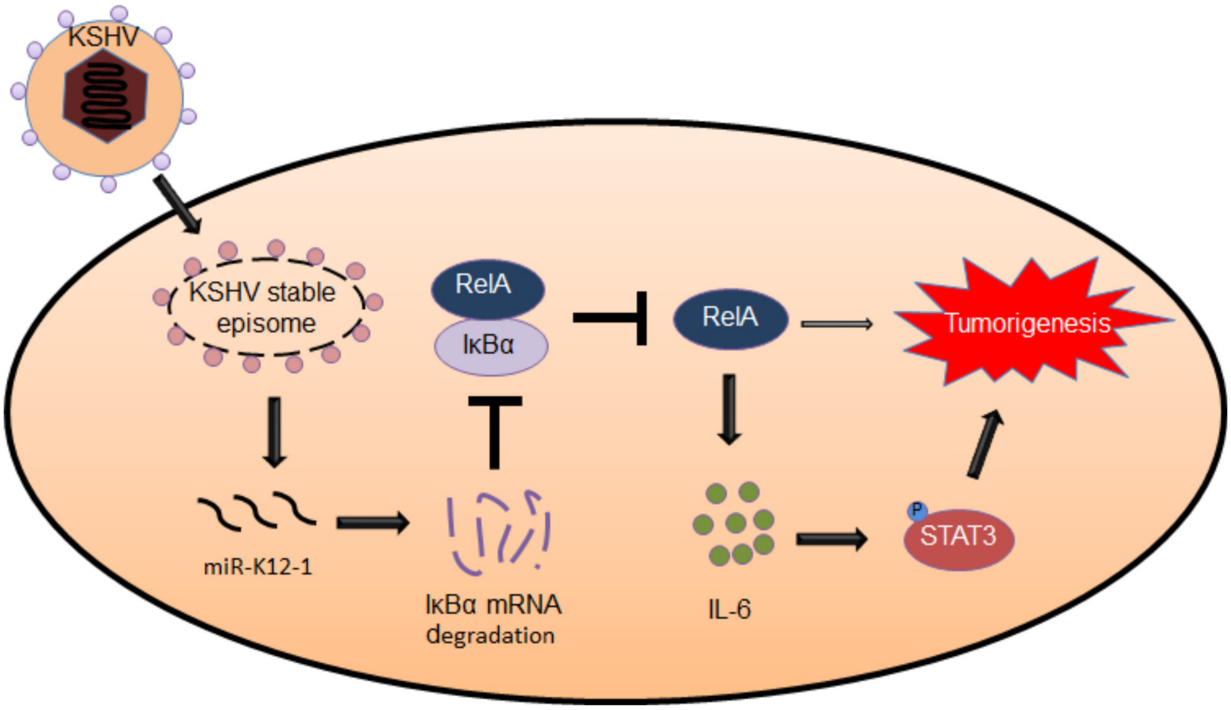

Supplementary Figure S9: Modeling of miR-K12-1/IκBα/NF-κB/IL-6/STAT3 signaling in KSHV tumorigenesis. NF-κB and STAT3 function as dimers but are indicated only by single protein.

Supplementary Table S1: target sites of shRNAs

Gene	Accession number	Target sequence
Human RelA	NM_021975.3	GGACATATGAGACCTTCAAGA
Human IL-6	NM_000600.3	AGACATGTAACAAGAGTAA
Human STAT3	NM_139276.2	GCACAATCTACGAAGAATCAA
Rat IL-6	NM_012589.2	GCTTCCAAACTGGATATAACC
Rat STAT3	NM_012747.2	GCTGCACCTGATCACCTTTGA

Supplementary Table S2: primer pairs used for real-time PCR

Gene	Accession number	Primer pairs	
		Forward (5′ to 3′)	Reverse (5′ to 3′)
GAPDH	NM_002046.3	CCGAGCCACATCGCTCAGACAC	GTGACCAGGCGCCCAATACGAC
IκBα	NM_020529.2	GGAGTACGAGCAGATGGTCAA	CCTTCACCTGGCGGATCAC
IL-6	NM_000600.3	TAGTGAGGAACAAGCCAGAGC	TGGGTCAGGGGTGGTTATTG
Survivin	NM_001168.2	TGACGACCCCATAGAGGAACA	CGCACTTTCTCCGCAGTTTC
Bcl-2	NM_000633.2	ATGTGTGTGGAGAGCGTCAACC	TGAGCAGAGTCTTCAGAGACAGCC
Bcl-xL	NM_138578.1	GAATGACCACCTAGAGCCTTGG	TGTTCCCATAGAGTTCCACAAAAG
Cyclin D1	NM_053056.2	CCGTCCATGCGGAAGATC	ATGGCCAGCGGGAAGAC
STAT3	NM_139276.2	CTAGATCGGCTAGAAAACTGGATAACGTC	ATGGGGTCCCCTTTGTAGGAAACT
RelA	NM_021975.3	CCAGACCAACAACAACCCCT	GATCTTGAGCTCGGCAGTGT
U6 snRNA	X59362.1	CTCGCTTCGGCAGCACA	AACGCTTCACGAATTTGCGT
miR-K12-1 reverse-transcription		GTCGTATCCAGTGCAGGGTCCGAGGTATTCGCACTGGATACGACGCTTAC	
miR-K12-1		CGCGCATTACAGGAAACTGGG	GTGCAGGGTCCGAGGT
